# Synthesis and Properties of a Novel Environmental Epoxidized Glycidyl Ester of Ricinoleic Acetic Ester Plasticizer for Poly(vinyl chloride)

**DOI:** 10.3390/polym9120640

**Published:** 2017-11-29

**Authors:** Jie Chen, Ke Li, Yigang Wang, Jinrui Huang, Xiaoan Nie, Jianchun Jiang

**Affiliations:** Institute of Chemical Industry of Forestry Products, Chinese Academy of Forestry, National Engineering Laboratory for Biomass Chemical Utilization, Key Laboratory of Biomass Energy and Material, Nanjing 210042, China; liketaiping@126.com (K.L.); wyg15063838536@126.com (Y.W.); huangjinruiahu@126.com (J.H.); bioenergy@126.com (J.J.)

**Keywords:** poly(vinyl chloride), renewable plasticizer, thermal stability, castor oil

## Abstract

A novel renewable plasticizer based on castor oil, epoxidized glycidyl ester of ricinoleic acetic ester (EGERAE), was synthesized and applied into Poly(vinyl chloride) (PVC) for the first time. Its molecular structure was characterized by FT-IR and ^1^H NMR. The effects of replacement of petroleum-based commercial plasticizer dioctyl phthalate (DOP) with EGERAE in poly(vinyl chloride) (PVC) films were researched. Thermal stability, dynamic mechanical property and mechanical properties of PVC films were investigated with thermogravimetric analysis (TGA), dynamic mechanical analysis (DMA) and tensile tests. The results indicated that this castor oil-based plasticizer was able to improve the thermal stability of PVC blends when partially of completely substituting for DOP. Furthermore, EGERAE endowed PVC resin with enhanced flexibility. In addition, the exudation, volatility and extraction resistance characteristics of plasticizers were researched. The degradation mechanism and possible interaction between EGERAE and PVC molecules in the plasticized system were also investigated.

## 1. Introduction

A plasticizer is defined as “a substance or a material incorporated into a plastic to increase its flexibility, workability or distensibility” by the International Union of Pure and Applied Chemistry Council (IUPAC) [1]. Plasticizers are one of the most common plastics additives [2]. During the last decade, around 1, 0.8 and 3.5 million tons of plasticizers were produced per year in Europe, North America and Asia, respectively [3]. Furthermore, the global annual market requirement for plasticizers is projected to rise to over 13.2 million tons per year until 2018 [3]. Phthalates, especially the dioctyl phthalate (DOP) and dibutyl phthalate (DBP), are the most commonly used plasticizers for poly(vinyl chloride) (PVC), and account for more than 80% of the global plasticizer market [4,5]. However, the usage of common plasticizers, especially phthalates, has sparked controversy due to their migration, as they are potentially toxic to humans and are susceptible to bioaccumulation in the environment. Restrictive regulations on the use of phthalates for flexible PVC products have been defined in many countries [6,7,8,9,10,11]. Consequently, with the growing interest in plasticizers with low toxicity and high migration resistance, and as an alternative to phthalates [12,13,14,15,16,17,18,19], more and more researchers are directing their attention to bio-based plasticizers made from vegetable oils [14,20,21,22].

Castor is a potential renewable resource that can derive environmentally sustainable plasticizers for plastics. The first plasticizers were used in the 1800s with natural camphor and castor oil [3]. In recent years, some kinds of castor oil-derived plasticizers have been reported. Mehta et al. [23,24] prepared two kinds of plasticizers by esterification reaction from ricinoleic acid. Feng et al. [25] researched the synthesis of phosphorus-containing plasticizers based on castor oil, and their application for flame retardancy of polyvinyl chloride. Jia et al. [26] compounded castor oil-based polyol ester plasticizers for PVC. In order to expand the utilization of castor oil, our research is directed toward the synthesis of new castor oil derivatives that are suitable as plasticizers for PVC. The functional epoxy groups in glycidyl esters are able to act as HCl scavengers and co-stabilizers, which can bring thermal stability to PVC [1]. The ester group in glycidyl ester allows specific interactions with the polymer, such as van der Waals and hydrogen bond interactions [1]. It could be expected that the conversion of hydroxy and carboxylic acid groups in ricinoleic acid to acetic ester and glycidyl ester, respectively, might derive a new green plasticizer for PVC. Due to the esters, epoxy groups and flexible alkyl chains, this plasticizer tends to endow PVC with good thermal stability and flexibility.

**Figure 1:**
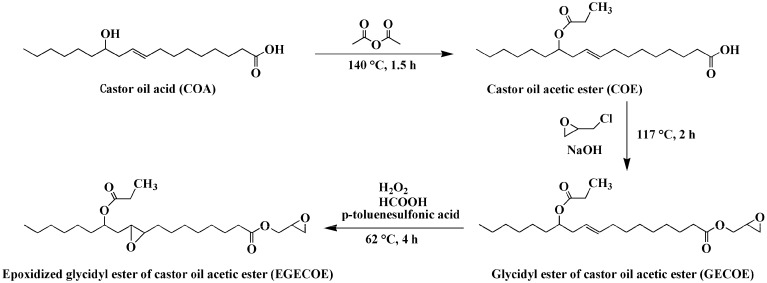
The synthesis routes of epoxidized glycidyl ester of ricinoleic acetic ester (EGERAE).

In this work, a novel renewable plasticizer derived from castor oil, epoxidized glycidyl ester of ricinoleic acetic ester (EGERAE), has been synthesized (Figure 1). The obtained plasticizer was incorporated into PVC as the main plasticizer, or as a secondary plasticizer alongside DOP. The plasticizing properties, such as thermal stability, dynamic mechanical property, mechanical properties and migration stability of PVC films were researched and compared to those of DOP. The degradation mechanism and possible interaction between EGERAE and PVC molecules in the plasticized system were also investigated.

## 2. Materials and Methods

### 2.1. Materials

Ricinoleic acid (97%), epichlorohydrin (99%), sodium hydroxide (97%), benzyltriethylammonium chloride (97%), dioctyl phthalate (99.5%) were obtained from Aladdin Chemical Reagent Co., Ltd., (Shanghai, China). *p*-Toluenesulfonic acid (98%), acetic anhydride (99%), formic acid (88%) and hydrogen peroxide (30%) were provided by Nanjing Chemical Reagent Co., Ltd. (Nanjing, China). Zinc stearate and calcium stearate were purchased from Changzhou Huaren Chemical Co., Ltd. (Changzhou, China) PVC (S-1000) was supplied by the Sinopec Qilu Co., Ltd. (Zibo, China).

### 2.2. Preparation of RAE

To a 100 mL flask equipped with a magnetic stirrer, reflux condenser and thermometer, ricinoleic acid (30.0 g) and acetic anhydride (10.2 g) were charged. Then the esterification reaction was continued at 140 °C for 1.5 h. After cooling to 60 °C, the reaction mixture was washed with distilled water to neutral. Finally, the water was removed through distillation under vacuum at 80 °C. Then the RAE was obtained.

### 2.3. Preparation of GERAE

RAE (22.0 g), epichlorohydrin (60.0 g) and benzyltriethyl ammoniumchloride (0.08 g) were added to a three-neck round bottom flask equipped with a thermometer, magnetic stirrer and reflux condenser. The reaction was continued for 2 h at 117 °C. Then, the mixture was cooled to 60 °C, and sodium hydroxide (2.6 g) was charged. After that, the reaction was continued at 60 °C for 3 h. The mixture was separated by brinell loopholes, and the solids were filtered. Then, the excess epichlorophydrin was recycled through distillation under vacuum. Finally, the GERAE was obtained.

### 2.4. Preparation of EGERAE

GERAE (50.0 g), formic acid (6.0 g) and *p*-toluenesulfonic acid (0.5 g) were added in a three-necked round bottom flask equipped with a thermometer, magnetic stirrer, and reflux condenser. When the temperature reached 50 °C, hydrogen peroxide (20.0 g) was added over 30 min. Then the reaction was continued for 4 h at 62 °C. After the reaction was complete, the mixture was filtered, and the filtrate was washed with a 2 wt % NaHCO_3_ and distilled water, respectively. After that, the organic phase was dried with anhydrous magnesium sulfate, and then filtered. Finally, a yellowish liquid product was obtained. The acid value and epoxy value of the EGERAE were 1.14 mg/g and 3.2%, respectively.

### 2.5. Preparation of Plasticized PVC Test Specimens

A series of plasticized PVC films with different plasticizers were prepared for comparison. Plasticizers, PVC powder and thermal stabilizers (Casalts/Zn salts = 3/1) were mixed using a mechanical mixer for 3 min at room temperature. Then the mixture was mixed at 165 °C for 5 min by using double-roller blending rolls (Zhenggong Co., Zhengzhou, China). Finally, PVC films with a thickness of 1 mm were obtained. The formulations of the PVC films are shown in Table 1.

### 2.6. Characterizations

Fourier transform infrared (FT-IR) spectra were confirmed by a Nicolet IS10 instrument (Thermo Fisher Scientific Inc., Waltham, MA, USA) in a range of 4000 to 500 cm^–1^ usin gan attenuated total reflectance method.

^1^H NMR spectra of the compounds in deuterated chloroform (CDCl_3_) were recorded on a Bruker ARX 300 spectrometer (Bruker Co., Karlsruhe, Germany) at room temperature.

Thermogravimetric analysis (TGA) was carried out in a 409PC thermogravimetric analyzer (Netzsch Co., Bavaria, Germany). Each specimen was scanned from ambient temperature to 600 °C under a nitrogen atmosphere at a heating rate of 10 °C/min.

Dynamic mechanical analysis (DMA) was measured using a DMA Q800 (TA Instruments, New Castle, DE, USA) in dual cantilever mode with a frequency of 1 Hz. The testing temperature was swept from –60 to 80 °C at a heating rate of 3 °C/min. For each specimen, replicated tests were performed in order to ensure the reproducibility of the data.

Tensile properties were measured using a SANS CMT-4303 universal testing machine (Shenzhen Xinsansi Jiliang Instrument Co., Shenzhen, China) according to ISO 527-2: 1993. The cross-head speed was set at 10 mm/min. All specimens were conditioned at 23 °C for 1 day prior to tensile testing. The tensile tests were recorded at 23 °C. Five specimens were prepared for each group to obtain an average value.

Exudation tests was studied by putting a PVC film between two pieces of filter paper. Then, this system was placed in a convection oven (Shanghai Suopu Instrument Co., Shanghai, China) for 48 h at 60 °C. The weight increments of the filter papers were measured. To obtain an average value, three samples were tested.

Volatility tests were determined by ISO 176:2005, the activated carbon method. The specimen was placed on the bottom of a metal container, and about 120 cm^3^ of activated carbon was spread over this specimen, before putting the lid was put on the container. The container was placed in the convection oven (Shanghai Suopu Instrument Co., Shanghai, China) at a temperature of 70 ± 1 °C. After 24 h, the container was removed from the oven and cooled at room temperature in a desiccator. The film was brushed and reweighed. The weight loss was measured before and after the heating. To obtain an average value, three samples were tested.

Extraction tests were based on ASTMD 1239-98. The PVC films were immersed in petroleum ether, soybean oil, distilled water, 30% (*w/v*) acetic acid, and 10% (*w/w*) sodium hydroxide at 23 ± 1 °C and 50 ± 5% relative humidity. The extracted PVC film was rinsed by distilled water and wiped up after 24 h. Then, each film was dried in a convection oven (Shanghai Suopu Instrument Co., Shanghai, China) for 24 h at 30 °C and reweighed. The weight loss before and after immersion was measured. Three samples were tested to get an average value.

## 3. Results and Discussion

### 3.1. Synthesis and Characterization

**Figure 2:**
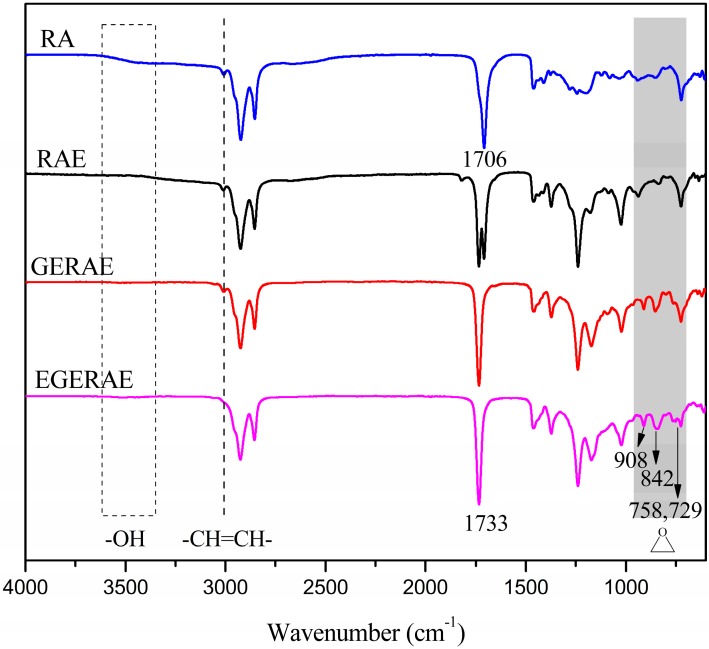
FT-IR spectra of RA, RAE, GERAE and EGERAE.

Figure 2 shows the FT-IR spectra of RA, RAE, GERAE and EGERAE. In the spectrum of RA, the broad absorption at around 3550–3250 cm^−1^ was due to the presence of C–H and O–H stretching vibrations. The peak at 1706 cm^–1^ was attributed to the carboxylic acid group. Compared to the FT-IR spectrum of RA, it can be seen that the absorption peaks of –OH group disappeared in the RAE spectrum. In the EGERAE spectrum, the absorption at around 1733 cm^–1^ corresponded to the carboxylic eater groups; furthermore, characteristic features of the glycidyl esters group were found at 910, 853 and 726 cm^–1^. In the spectrum of EGERAE, the absorption at around 3009 cm^–1^ attributed to the –CH=CH– disappeared. In addition, the epoxy groups peaks were observed at 908, 842, 958 and 729 cm^–1^. These characteristic peaks suggested that EGERAE had been obtained.

**Figure 3:**
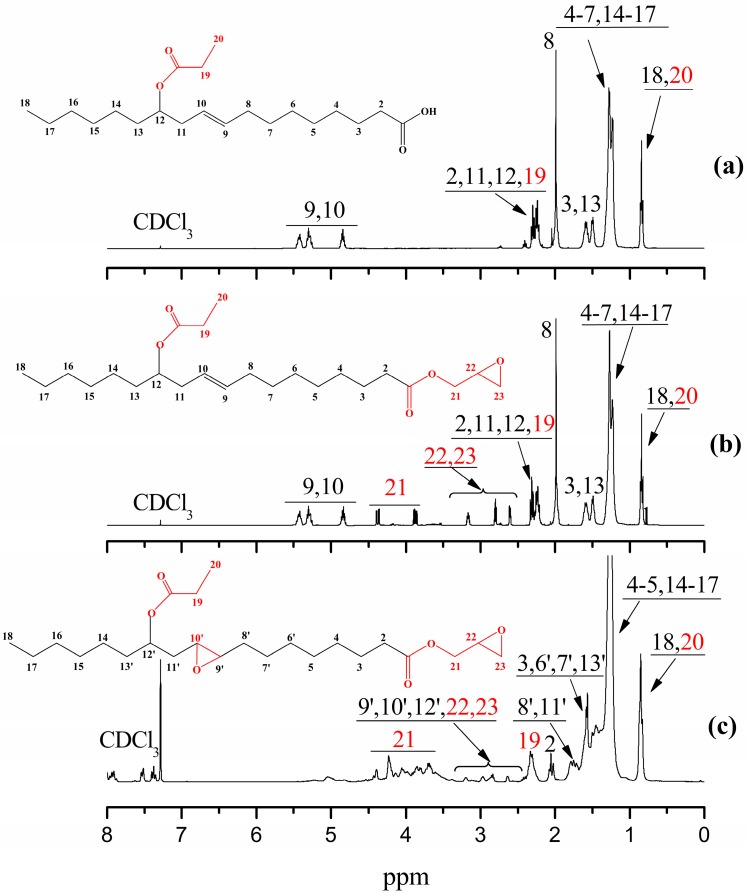
^1^H NMR spectra of (**a**) RAE, (**b**) GERA and (**c**) EGERAE.

Figure 3 displays the ^1^H NMR spectra of RAE, GERA and EGERAE. In Figure 3a, the peak at 0.91 ppm was an indication of methyl of number 18 and 20 carbon. The peaks at around 1.13–1.39 ppm (peaks 4–7 and 14–17) were signs of methylene. The peaks at 1.45–1.64 ppm were attributed to the hydrogens of –CH_2_–, in addition to the carboxyls of number 3 and 13 carbon. The chemical shifts around 1.95–2.41 ppm (peaks 2, 11, 12, 19, 8) corresponded to the methylene and methine protons attached to the double bond and –COOH groups. The multi peaks at 4.84–5.42 ppm were attributed to the hydrogens of CH=CH of number 9, 10 carbon. The new peaks at 2.62–4.38 ppm (peaks 21–23) were attributed to the protons of the glycidyl ester group that appeared in Figure 3b, indicating that an esterification reaction of RAE with epichlorohydrin had occurred. In Figure 3c, the peaks of number 9, 10 carbon at around 5 ppm disappeared. The new peaks at around 2.59–3.14 ppm were the signs of the protons of the epoxy groups, which implied that GERAE had transformed into EGERAE.

### 3.2. Thermal Stability

**Figure 4:**
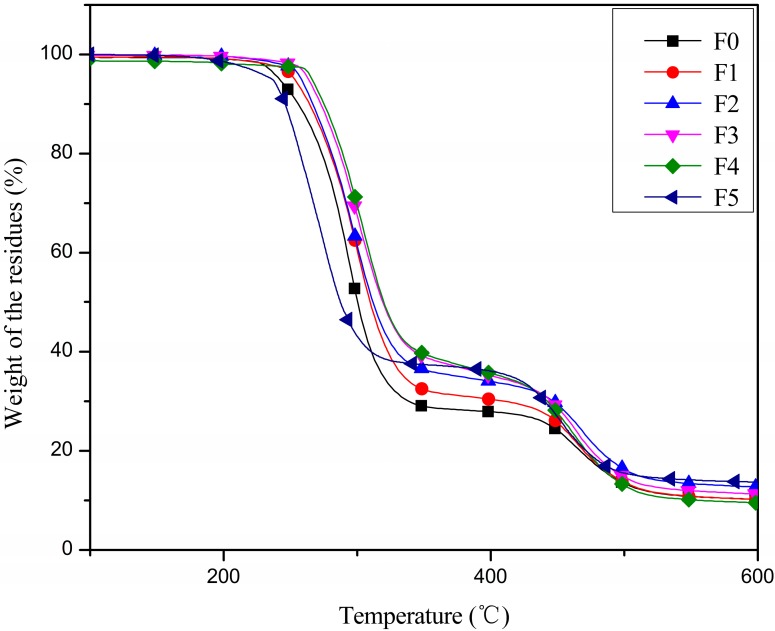
TGA curves of plasticized PVC films.

**Figure 5:**
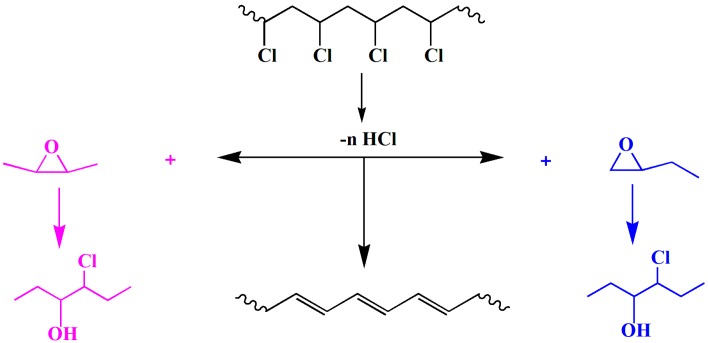
Degradation mechanism of PVC with glycidyl ester plasticizer.

The TGA curves of the PVC films heated in nitrogen at a rate of 10 °C/min were evaluated, and the results are shown in Figure 4. The thermal degradation data, including initial decomposition temperature (*T*_i_), 10% and 50% mass-loss temperatures (*T*_10_ and *T*_50_), and residue at 300 and 400 °C (*R*_300_, *R*_400_), are summarized in Table 2. It can be observed that the initial degradation of film plasticized with pure DOP (film F0) occurred at 262.2 °C, while EGERAE displayed initial decomposition at 271.8 °C. Furthermore, the same PVC, handled the same way, was used for direct comparisons between the results of EGERAE and the results of epoxidized fatty acid methyl ester (EFAME), published in [27]. Compared with the TGA results of PVC film F5 plasticized with EFAME, the *T*_i_ of film F4 increased from 248.4 [27] to 271.8 °C, which might be due to the molecular weight of EGERAE (412.5) being higher than that of EFAME (about 312). It was also shown that the *T*_10_ and *T*_50_ of PVC films increased when DOP was gradually replaced with EGERAE. Compared with film F0, the *T*_10_ and *T*_50_ of film F4 increased from 255.5 and 300.5 to 275.8 and 320.8 °C, respectively. This is mainly due to the glycidyl esters group and the epoxy group of EGERAE, which are able to scavenge for HCl and delay thermal degradation (Figure 5) [28,29,30]. The results indicate that the introduction of EGERAE is able to better enhance the thermal stabilities of PVC films than DOP or EFAME.

### 3.3. Dynamic Mechanical Analysis

**Figure 6:**
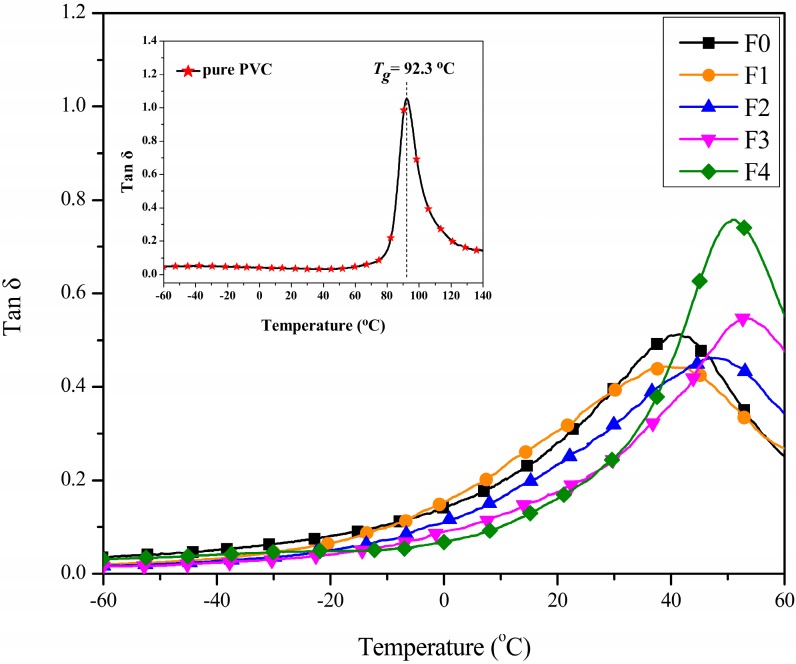
DMA curves for the PVC films with different plasticizers.

**Figure 7:**
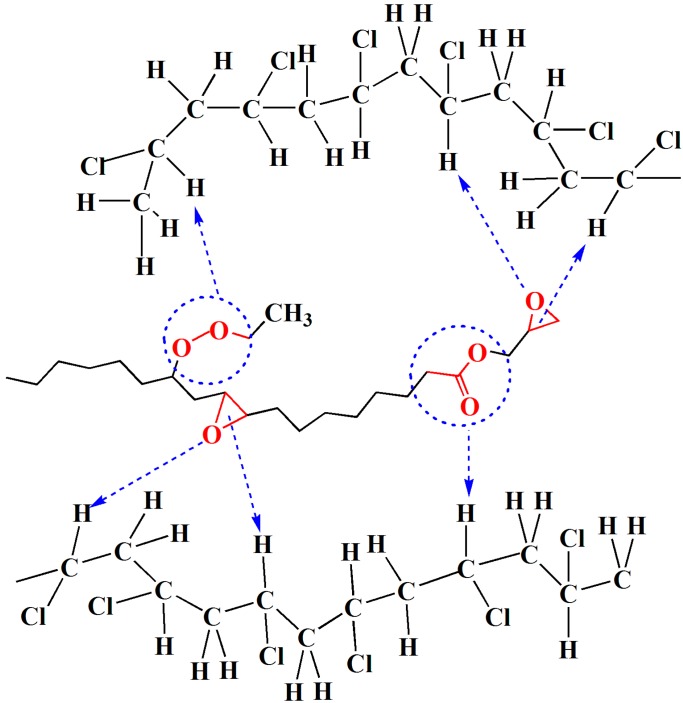
Possible interaction between EGERAE and PVC molecules in the plasticized system.

The dynamic mechanical properties of the PVC films were investigated using DMA technology. Figure 6 shows the loss factor (tan δ) curves of the PVC films with different plasticizers. Only one peak could be seen in each curve, suggesting that the blend was a homogenous material, and that the plasticizer had good compatibility with PVC [3,31]. Glass transition temperature (*T*_g_) was determined as the temperature at the maximum of the tan δ curve, and is presented in Table 2. The *T*_g_ value of films F0-F4 were 41.5, 39.2, 46.9, 53.1 and 51.0 °C, respectively, which were far below the value of 92.3 °C for pure PVC. It can be seen that the *T*_g_ of film F1 (with 10 phr EGERAE) is lower than that of film F0. This can be explained by the fact that the long alkyl chain and epoxy polar groups of EGERAE lubricated and interacted with the polar fraction of the PVC molecule, decreasing the crosslinking of PVC-PVC, increasing the free volume of the polymer’s amorphous region (as shown in Figure 7), and resulting in a decrease in *T*_g_. With the increase of EGERAE content, tan δ value peaks shifted towards higher temperatures, and the corresponding *T*_g_ increased [32]. This might have been caused by the chemical structure of EGERAE [33,34]. Due to the higher molecular weight of EGERAE (412.5) compared with DOP (391), the plasticizing effect of the plasticizer on PVC would be correspondingly reduced when DOP was replaced with more than 10 phr EGERAE. However, the addition of EGERAE increased the damping capability, which led to taller and narrower tan δ peaks than those of film F0.

### 3.4. Mechanical Properties

Table 3 shows the results for tensile strength, elongation at break, and modulus of elasticity for PVC films with different plasticizers. It can be observed that PVC films plasticized with EGERAE (F1-F4) had similar tensile strength, better elongation at break, and higher modulus of elasticity properties than the film F0. After adding 0 to 30 phr EGERAE, the elongation at break of film F0-F3 generally increased from 256.9% to 308.4%, respectively. When 20 phr EGERAE was added, the elongation at break of the PVC film was 337.9%, which was higher than that of the PVC film plasticized with EFAME (318.9%) [27]. However, when the concentration of EGERAE was increased further, to 40 phr, the elongation at break was similar to that of film F0. This is due to the shielding effect and lubricity of the long alkyl chain structure of the EGERAE and EFAME. It can also be seen from Table 3 that the tensile strength and modulus of elasticity increased with the increase in EGERAE concentration, which suggested the enhancement of the strength of the PVC blends. However, the film F1, in comparison with F0, had better elongation at break and similar tensile strength and modulus of elasticity, implying that the influence of flexible alkyl chains might be balanced with that of the rigidity groups when 10 phr EGERAE was added. This result was consistent with the Bargellini’s research, which suggested that tensile strength is connected with the magnitude of the plasticizer’s polarity and the plasticizer’s proportions [35,36]. It can be concluded that the introduction of EGERA Eis able to endow PVC resin with better flexibility and thus improve the strength of PVC compounds.

### 3.5. Exudation, Volatility and Extraction Resistance

**Figure 8:**
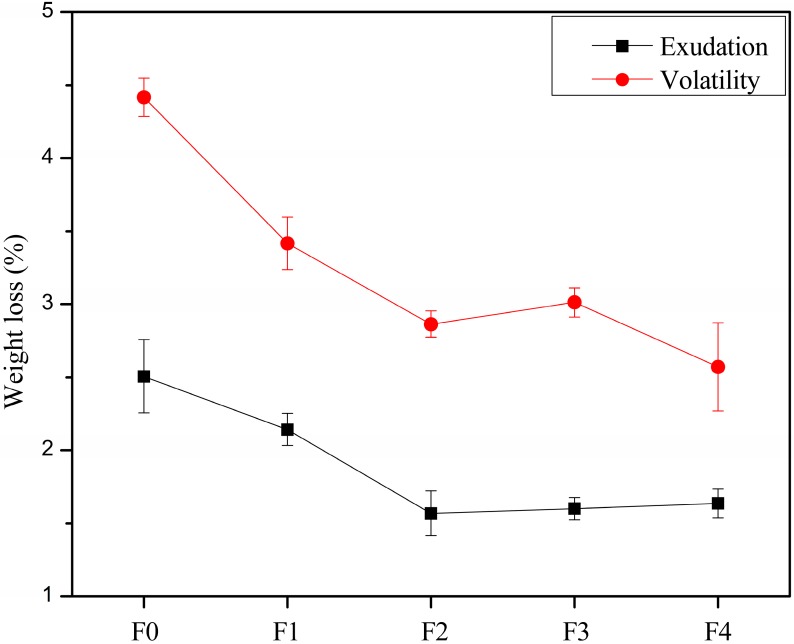
Weight losses of PVC films after exudation and volatilization testing.

The weight losses of the PVC films after exudation and volatility tests are shown in Figure 8. It can be seen that the exudation resistances of films F1–F4 were similar to film F0. On the other hand, the weight loss after volatility testing generally followed the order F0 > F1 > F2 > F3 > F4, indicating the volatilization loss generally decreased with an increase in EGERAE content.

**Figure 9:**
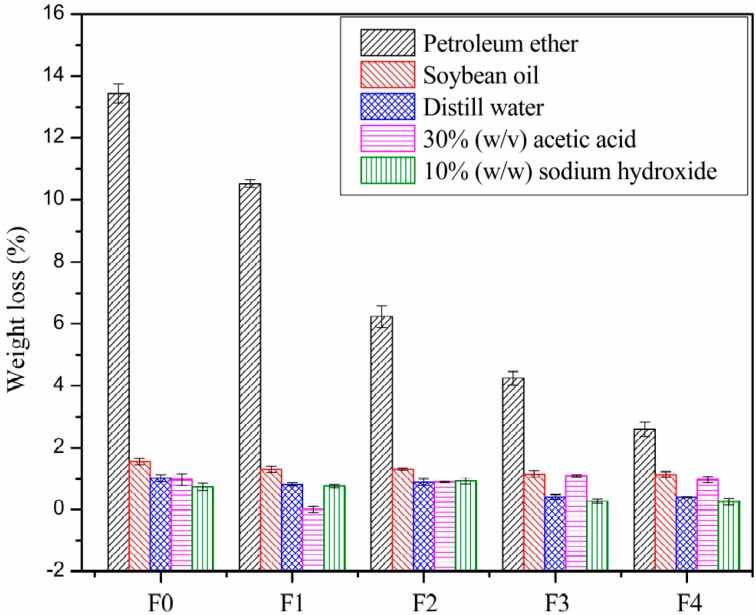
Weight losses of PVC films after extraction testing.

The weight losses of the PVC films by exudation in petroleum ether, soybean oil, distill water, 30% (*w/w*) acetic acid and 10% (*w/w*) NaOH are shown in Figure 9. The PVC films showed different migration resistances during extraction testing. When leached in petroleum ether, all of the plasticizers showed poorer migration resistance than in the other solvents. It could be clearly observed that the migration resistance in petroleum ether was improved with the substitution of EGERAE into DOP. Furthermore, all of the films showed poorer extraction resistance in soybean oil than in distilled water, acid and alkali. This is mainly due to the plasticizers being organic solvents. In addition, with the substitution of EGERAE into DOP, the resistance of extraction by 30% (*w/w*) acetic acid and 10% (*w/w*) NaOH was similar to that of the film using pure DOP.

## 4. Conclusions

A novel renewable plasticizer EGERAE was synthesized and applied in PVC blends to evaluate its effects on thermal properties, mechanical properties and migration stabilities of PVC films. The effects of EGERAE partially or completely replacing DOP were investigated. The results of TGA indicated that this castor oil-based plasticizer was able to improve the thermal stability of PVC blends. When DOP was completely substituted with EGERAE, the *T*_i_, *T*_10_, *T*_50_, *R*_300_ and *R*_400_ increased by 9.6, 20.3, 20.3 °C, 19.1%, and 7.9%, respectively. Mechanical tests were carried out, and the results suggested EGERAE could endow PVC resin with enhanced flexibility. In addition, with the substitution of DOP by EGERAE, PVC films exhibited better migration stability. The degradation mechanisms and possible interactions in the plasticized system were studied. Consequently, this castor oil-based plasticizer is a potential alternative plasticizer in environmentally friendly plastic materials.

## Figures and Tables

**Table 1:** Formulation of the poly(vinyl chloride) (PVC) films.

Component (phr)	F0	F1	F2	F3	F4
Total plasticizer content	40.0	40.0	40.0	40.0	40.0
EGERAE content	0.0	10.0	20.0	30.0	40.0
DOP content	40.0	30.0	20.0	10.0	0.0
EFAME content	0.0	0.0	0.0	0.0	0.0
Thermal stabilizers content	2.0	2.0	2.0	2.0	2.0

**Table 2:** Thermal properties of PVC films with different plasticizers.

Sample	*T*_g_ (°C)	*T*_i_ (°C)	*T*_10_ (°C)	*T*_50_ (°C)	*R*_300_ (%)	*R*_400_ (%)
F0	41.5	262.2	255.5	300.5	49.6	27.9
F1	39.2	263.7	265.7	310.7	59.6	30.5
F2	46.9	261.6	268.2	313.2	60.6	34.1
F3	53.1	267.1	273.1	320.6	66.8	35.4
F4	51.0	271.8	275.8	320.8	68.7	35.8

**Table 3:** Results obtained from tensile measurements.

Sample	Tensile Strength (MPa)	Elongation at Break (%)	Modulus of Elasticity (MPa)
F0	5.0 ± 0.38	256.9 ± 23.77	2.4 ± 0.09
F1	5.6 ± 0.32	318.8 ± 20.27	2.5 ± 0.24
F2	6.0 ± 0.16	337.9 ± 21.47	9.6 ± 4.78
F3	5.9 ± 0.55	308.4 ± 19.59	27.5 ± 1.76
F4	6.8 ± 0.59	264.7 ± 13.40	57.5 ± 6.97
